# Supplementary material for: End Sequence Analysis Toolkit (ESAT) expands the extractable information from single-cell RNA-seq data
Source: Genome Res. 2016 Oct;26(10):1397–410. doi: 10.1101/gr.207902.116 (PMC5052061; doi:10.1101/gr.207902.116)
Supplement: Supplemental Material [file supp_gr.207902.116_Supp_Materials.tar.gz › ESAT-esat_v0.1/doc/html/classbroad_1_1core_1_1math_1_1_math_util.html]

ESAT: broad.core.math.MathUtil Class Reference


ESAT


- Main Page
- Related Pages
- Packages
- Classes
- Files

- Class List
- Class Index
- Class Hierarchy
- Class Members

All Classes Namespaces Files Functions Variables Enumerator Pages

- broad
- core
- math
- MathUtil

Public Member Functions |
Static Public Member Functions |
List of all members

broad.core.math.MathUtil Class Reference

|  |  |
| --- | --- |
| Public Member Functions | |
| static< T > List< T > | shuffle (List< T > original) |
|  | |

|  |  |
| --- | --- |
| Static Public Member Functions | |
| static double[][] | roots (double[] coefs) |
|  | |
| static double | log2 (double val) |
|  | |
| static double | max (Collection<?extends Number > list) |
|  | |
| static double[] | shuffle (double[] original) |
|  | |
| static double | hypot (double a, double b) |
|  | |
| static long | binomialCoefficient (int n, int k) |
|  | |
| static long | factorial (int n) |
|  | |
| static boolean | closeTo1 (double d) |
|  | |

## Member Function Documentation

|  |  |  |  |
| --- | --- | --- | --- |
| static long broad.core.math.MathUtil.binomialCoefficient | ( | int | *n*, |
|  |  | int | *k* |
|  | ) |  |  |

static

The binomial coefficient n choose k

Parameters
:   |  |  |
    | --- | --- |
    | n | n |
    | k | k |

Returns
:   n choose k

Here is the call graph for this function:

|  |  |  |  |  |  |
| --- | --- | --- | --- | --- | --- |
| static boolean broad.core.math.MathUtil.closeTo1 | ( | double | *d* | ) |  |

static

|  |  |  |  |  |  |
| --- | --- | --- | --- | --- | --- |
| static long broad.core.math.MathUtil.factorial | ( | int | *n* | ) |  |

static

Factorial function

Parameters
:   |  |  |
    | --- | --- |
    | n | n |

Returns
:   n!

Here is the caller graph for this function:

|  |  |  |  |
| --- | --- | --- | --- |
| static double broad.core.math.MathUtil.hypot | ( | double | *a*, |
|  |  | double | *b* |
|  | ) |  |  |

static

sqrt(a^2 + b^2) without under/overflow.

|  |  |  |  |  |  |
| --- | --- | --- | --- | --- | --- |
| static double broad.core.math.MathUtil.log2 | ( | double | *val* | ) |  |

static

|  |  |  |  |  |  |
| --- | --- | --- | --- | --- | --- |
| static double broad.core.math.MathUtil.max | ( | Collection<?extends Number > | *list* | ) |  |

static

|  |  |  |  |  |  |
| --- | --- | --- | --- | --- | --- |
| static double [][] broad.core.math.MathUtil.roots | ( | double[] | *coefs* | ) |  |

static

Returns the roots of a polynomial with given coefficients.

Parameters
:   |  |  |
    | --- | --- |
    | coefs | - Polynomial coefficients in increasing order (e.g. the polynomial is of the form coefs[0] + coefs[1]\*X + coefs[2]\*X^2 + ... |

Returns

|  |  |  |  |  |  |
| --- | --- | --- | --- | --- | --- |
| static double [] broad.core.math.MathUtil.shuffle | ( | double[] | *original* | ) |  |

static

Here is the caller graph for this function:

|  |  |  |  |  |  |
| --- | --- | --- | --- | --- | --- |
| static<T> List<T> broad.core.math.MathUtil.shuffle | ( | List< T > | *original* | ) |  |

Here is the call graph for this function:

---

The documentation for this class was generated from the following file:

- java/broad/core/math/MathUtil.java


---

Generated on Fri Aug 15 2014 15:10:53 for ESAT by  

 1.8.7
